# Data processing pipeline for serial femtosecond crystallography at SACLA^1^

**DOI:** 10.1107/S1600576716005720

**Published:** 2016-04-18

**Authors:** Takanori Nakane, Yasumasa Joti, Kensuke Tono, Makina Yabashi, Eriko Nango, So Iwata, Ryuichiro Ishitani, Osamu Nureki

**Affiliations:** a Department of Biological Sciences, Graduate School of Science, The University of Tokyo, 2-11-16 Yayoi, Bunkyo, Tokyo 113-0032, Japan; b Japan Synchrotron Radiation Research Institute, 1-1-1 Kouto, Sayo-cho, Sayo-gun, Hyogo, 679-5198, Japan; c RIKEN SPring-8 Center, 1-1-1 Kouto, Sayo-cho, Sayo-gun, Hyogo, 679-5148, Japan; d Department of Cell Biology, Graduate School of Medicine, Kyoto University, Yoshidakonoe-cho, Sakyo-ku, Kyoto, 606-8501, Japan

**Keywords:** serial femtosecond crystallography, real-time processing, spot finding, parallelization, SACLA

## Abstract

A data processing pipeline for serial femtosecond crystallography at SACLA was developed, based on *Cheetah* [Barty *et al.* (2014). *J. Appl. Cryst.***47**, 1118–1131] and *CrystFEL* [White *et al.* (2016). *J. Appl. Cryst.***49**, 680–689]. The original programs were adapted for data acquisition through the SACLA API, thread and inter-node parallelization, and efficient image handling. The pipeline consists of two stages: The first, online stage can analyse all images in real time, with a latency of less than a few seconds, to provide feedback on hit rate and detector saturation. The second, offline stage converts hit images into HDF5 files and runs *CrystFEL* for indexing and integration. The size of the filtered compressed output is comparable to that of a synchrotron data set. The pipeline enables real-time feedback and rapid structure solution during beamtime.

## Abstract 2

A data processing pipeline for SACLA was developed, based on *Cheetah* and *CrystFEL*. Real-time analysis and rapid structure solution were enabled.

## 1. Introduction   

Serial femtosecond crystallography (SFX) utilizes ultra-short but intense X-ray pulses, generated by X-ray free-electron laser (XFEL) facilities, to collect diffraction images from tens of thousands of microcrystals (Kirian *et al.*, 2010 ▸). It has been applied to structure determinations of radiation-damage-sensitive microcrystals (e.g. Redecke *et al.*, 2013 ▸; Kang *et al.*, 2015 ▸) and time-resolved studies of light-triggered reactions (Tenboer *et al.*, 2014 ▸; Barends *et al.*, 2015 ▸). Although recent developments in sample injection methods (Sierra *et al.*, 2012 ▸; Weierstall *et al.*, 2014 ▸; Sugahara *et al.*, 2015 ▸) and data processing algorithms (White, 2014 ▸; White *et al.*, 2016 ▸; Sauter, 2015 ▸; Uervirojnangkoorn *et al.*, 2015 ▸; Ginn *et al.*, 2015 ▸) have reduced the number of crystals, the amount of time and the quantity of data necessary for structure solution, thousands to tens of thousands of high-resolution diffraction patterns are still required to obtain an accurate data set.

During SFX beamtime, which typically lasts for 12–48 h at SACLA, millions of images are collected (see *Example* [Sec sec9] section below for actual numbers). However, usually more than half of the XFEL pulses miss the crystals, and some crystals are too small or too disordered to give useful diffraction. Thus, most of the recorded images do not contain high-resolution diffraction patterns. Owing to the huge numbers of images, it is impractical for humans to inspect all of them. Thus, an automated data processing system is essential for successful SFX experiments.

An important aspect of such a system is to provide rapid feedback to experimenters. Analyses of hit rates provide valuable information to make strategic decisions. For example, if the crystal density or resolution of the current crystallization batch is low, one might want to abandon the current sample and look for a better one for optimal use of precious beamtime. Real-time feedback is also essential for the alignment of the X-ray beam and the sample stream, especially when the stream is small, to minimize sample consumption and background scattering. Similarly, an analysis of detector saturation is necessary to determine the optimal attenuator thickness.

Another goal of such a system is image filtering and data conversion. Data sets should be filtered to separate the good diffraction patterns from the many bad images, because bad images make the data set large, leading to longer processing time and increased storage costs. Filtered images and associated metadata must be converted to a format suitable for subsequent processing.

Finally, these processes should be performed with minimum human intervention. Routine tasks common to all data sets should be automated, so that users can focus on making decisions and project-specific analyses. Automation also serves to reduce human errors.

Toward these ends, several pre-processing programs have been developed, including *Cheetah* (Barty *et al.*, 2014 ▸), *CASS* (Foucar *et al.*, 2012 ▸), *cctbx.xfel* (Sauter *et al.*, 2013 ▸) and *psana* at LCLS (Damiani *et al.*, 2016 ▸). We have adapted *Cheetah* and *CrystFEL* (White *et al.*, 2012 ▸, 2013 ▸, 2016 ▸) for the experimental and computational environments at SACLA, and developed a graphical user interface (GUI) to facilitate job submission and real-time monitoring. In this article, we report these developments, which might be applicable to serial crystallography experiments at other facilities.

## 2. Online and offline API   

**Figure 1:**
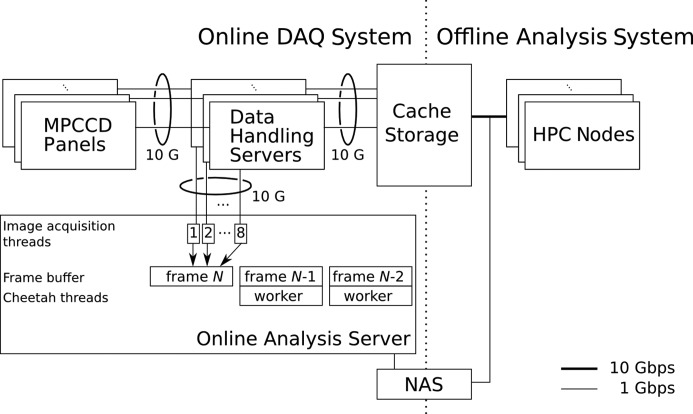
Architecture of the data processing environment at SACLA. The online pipeline runs on the online analysis server. Eight image acquisition threads retrieve image data from the corresponding data handling servers and fill the frame buffer (shown by arrows). Once completed, a *Cheetah* worker thread is dispatched for each frame. For simplicity, only two worker threads are drawn. The offline pipeline runs on HPC nodes.

The computing environment and data acquisition (DAQ) system at SACLA were described previously (Joti *et al.* 2015 ▸). Here we summarize what is relevant to this paper (Fig. 1 ▸). Raw data from eight multi-port charge coupled device (MPCCD) sensor modules (Kameshima *et al.*, 2014 ▸) are captured by frame grabbers and transferred to data handling servers. These servers temporarily buffer images and write all of them to the cache storage. Online analysis servers can intercept data from the data handling servers for real-time analysis. As discussed below, low-level filtering and programs using the SACLA online API can be executed only on these servers. These servers constitute the online part of the SACLA DAQ system. The offline analysis is performed on SACLA HPC nodes. These nodes can access the cache storage, the metadata database (DB) and the long-term archive storage, but not the data handling servers. This separation ensures that the online system can always achieve data collection at 60 Hz, independently of the loads on the offline system.

Image data are accessible through the SACLA API. Metadata, such as shot-by-shot spectra and photodiode readouts in pump–probe experiments, are also available through the API. Metadata are associated with an image by a tag number, a unique 64 bit serial number associated with each XFEL pulse. The API comes in two versions: online API and offline API.

The online API is used to intercept data from memory on the data handling servers. All detector frames can be extracted at 60 Hz with a latency of a few tens of milliseconds. The online API is available only at the online analysis servers, which have direct connections to the data handling servers. The online analysis server has limited output bandwidth (<1 Gbps) to a network attached storage (NAS) device, which is mounted from both the online analysis servers and the HPC nodes. Thus, it cannot write processed images in real time, but only processing logs.

The offline API targets images and metadata in the storage. They can be in the cache storage or long-term archive storage, depending on the time after data collection. Data access is encapsulated by the API, so user programs (including this pipeline) are unaware of the actual location. The offline API has limited throughput (at most 10 Hz for eight sensors per single thread) owing to IO bottlenecks. Since the offline API can only read images from completed runs, a run must finish before processing. This imposes a latency of about three minutes (time to collect 5150 images in a run at 30 Hz).

## 3. Online and offline pipelines   

**Figure 2:**
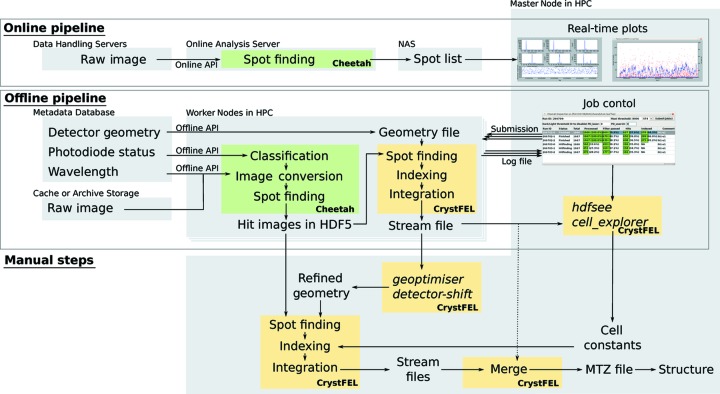
Typical flow of data in the pipeline. The online pipeline finds spots on images and provides real-time feedback on hit rates and detector saturation. The offline pipeline runs spot finding again and outputs hit images in the HDF5 format. The images are then processed by *CrystFEL*. In time-resolved experiments, excited and non-excited images are classified by the photodiode status. Users optimize parameters based on the pipeline output and re-run *CrystFEL* before final merging. If the refined parameters are known beforehand, then they can be used in the pipeline and the stream files from the pipeline can be immediately merged (dotted line).

Since the online and offline APIs have specific limitations, we run data processing in two stages (Fig. 2 ▸). The first stage, the online pipeline, is based on the online API. The purpose is to run spot finding on all images by using *Cheetah*, which provides real-time feedback on hit rates and detector saturation. Owing to limited output bandwidth, only the spot finding results, but not the images, are written to the NAS. The second stage, the offline pipeline, which uses the offline API, runs the spot finding again, converts the hit images into an HDF5 file and runs *CrystFEL*. In time-resolved studies, it also classifies excited and non-excited images, on the basis of photodiode readouts. Details of these steps are described in the following sections.

**Figure 3:**
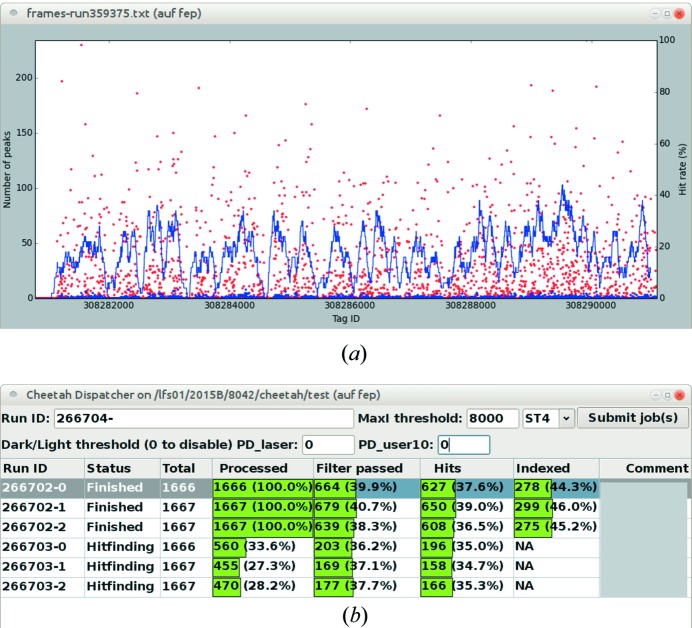
(*a*) Screenshot of the monitor for the real-time pipeline. The number of spots in each image is plotted in red, while the number of saturated spots is plotted in blue. The hit rate is represented by the blue line. (*b*) Screenshot of the GUI for the offline pipeline.

The GUI was developed using the *wxPython* (Talbot, 2000 ▸) library. The monitor for the first, online stage provides a real-time plot of numbers of spots and saturated spots and hit rates (Fig. 3 ▸ *a*). The GUI for the second, offline stage, called *Cheetah Dispatcher*, allows users to start the pipeline for specified runs. It also monitors the progress of data collection and automatically submits pipeline jobs as soon as a run has been completed. The results from jobs are displayed in a table (Fig. 3 ▸ *b*), in which the columns include numbers of frames, hits and indexed lattices. These numbers are updated every few seconds. By right-clicking rows, users can calculate the sums of these numbers to check whether the planned numbers (*e.g.* 10 000) of indexable images have been collected, and launch *hdfsee* or *cell_explorer* to examine the outputs.

**Figure 4:**
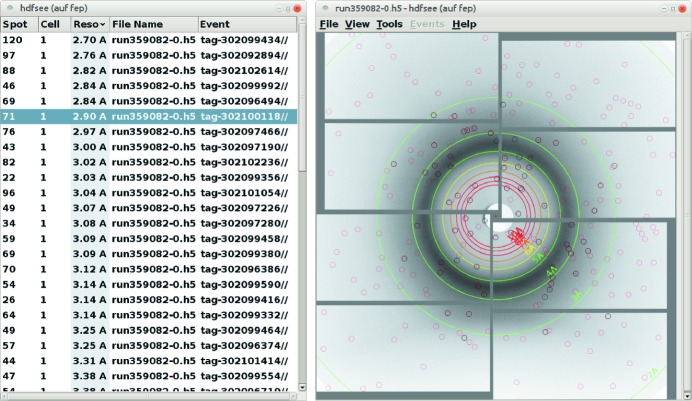
Screenshot of the extended *hdfsee* viewer. A table of images in a stream file is displayed on the left. On the right, spots identified by *CrystFEL* are circled in black, while predicted and integrated spots are circled in red.

To facilitate the examination of diffraction images, the *hdfsee* viewer in the *CrystFEL* suite was enhanced (Fig. 4 ▸). The enhanced version reads spot finding and integration results from a *CrystFEL* stream file and shows them in a table, which can be sorted by the number of spots or resolution estimates. Strong spots detected by the spot finding routine and integrated (predicted) spots are marked in different colours. This helps users to adjust the spot finding parameters and confirm the validity of the mosaicity model.

## 4. Image conversion   

The SACLA online and offline APIs provide MPCCD image data after static, dynamic and leakage calibrations (Kameshima *et al.*, 2014 ▸). Our pipeline further applies several image conversions.

In a typical SFX experiment at SACLA, the first 150 frames of each run are dark images collected while the shutter is closed, followed by 5000 exposed images. The average of the dark images is subtracted from the exposed images. Next, pixel values are rescaled so that ten units correspond to one photon. This conversion is performed as
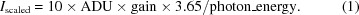
Here, 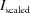 is the output value, ADU is the pixel value from the API, gain is the number of electrons per detector unit, 3.65 is the energy (in eV) to create an electron–hole pair in the detector silicon and photon_energy (in eV) is the energy of incoming X-ray photons. The sensor-specific gain is measured by the detector team [see the *Calibration* section in the paper by Kameshima *et al.* (2014 ▸)] and is available from the SACLA API. This normalization makes the spot finding parameters less sensitive to the photon energy. Finally, pixel values are converted from 32 bit floating point numbers to 16 bit integers.

The MPCCD used in SFX experiments consists of eight sensor modules (512 × 1024 pixels each). In the memory, images from the eight modules are stacked vertically into an array with 512 × 8192 elements. This does not reflect the physical arrangement (metrology) of the eight modules in space. Thus, subsequent programs need the detector geometry information as metadata, in addition to the pixel values. This is obtained from the SACLA API, and geometry files for *Cheetah* and *CrystFEL* are generated by the pipeline. We are also developing a *dxtbx* (Parkhurst *et al.*, 2014 ▸) module, which enables data processing by *DIALS* (Waterman *et al.*, 2013 ▸) and *cctbx.xfel* (Sauter *et al.*, 2013 ▸).

## 5. Hit finding   

For hit finding in *Cheetah*, algorithm 6 is used. It first binarizes the image by thresholding and generates spot candidates by decomposing strong pixels into connected components. Candidates are further filtered by the area and signal-to-noise ratio. When the number of accepted spots is above a threshold (typically 20), the image is retained as a hit. Images with fewer spots are of low resolution and can be discarded without affecting the data set quality.

Although the SACLA DAQ system has a low-level filtering (LLF) function, its applicability to SFX turned out to be very limited. As described by Joti *et al.* (2015 ▸), LLF calculates the maximum or average pixel value within the region of interest (ROI) of an image and stores it in the metadata DB. Since images in SFX experiments often contain strong ring-shaped scattering from the carrier medium, such as grease and lipidic cubic phase, it is difficult to distinguish protein diffraction patterns from others. The exclusion of such rings from the ROI is essential but tricky. Since LLF calculations are performed in parallel with data collection and cannot be reprocessed, the ROI must be defined before the data collection. Even with an adequate ROI, the LLF values do not necessarily show a bimodal distribution and finding a good threshold is difficult. Thus, we decided to use LLF only to reject obvious blank frames, by setting the threshold to a very low value (∼50 photons), and to rely on *Cheetah* for hit finding. We note that LLF may be useful for other experiments at SACLA, such as single-particle imaging.

## 6. Image output   

Each *Cheetah* job writes all hit images to a single file in the HDF5 format (The HDF Group, 1997 ▸). As compared to single-event (one file per image) HDF5 files produced by earlier versions of *Cheetah*, this multi-event HDF5 file has a smaller file system overhead. We designed and implemented a SACLA-specific HDF5 structure instead of the CXIDB format (Maia, 2012 ▸). In the CXIDB format, multiple images are stored in a three-dimensional array. In contrast, we create an HDF5 group named tag-*N* (*N* is the tag number) for each image, which contains the image itself and associated metadata (*e.g.* the mean of the pulse spectrum). The rationale is that the index of an image within the three-dimensional array is variable when a user re-runs *Cheetah*, while an HDF5 group name based on the image tag number is a unique constant.

Image data are compressed by the *deflate* algorithm (Deutsch, 1996 ▸). The filter mechanism in the HDF5 library allows transparent compression; manual decompression is unnecessary and no temporary files are created during decompression. Although the byte-offset filter in *CBFlib* (Ellis & Bernstein, 2006 ▸) might be faster and more effective, the *deflate* filter was chosen because the latter is distributed with the HDF5 library. Since users might want to copy pipeline outputs to their home institution for analysis by their own programs, the use of a standard, widely adopted compression algorithm is an advantage. Our benchmark tests showed that compression at level 5 reduces the file size by more than 50%, while the increase in the total run time is less than 5%.

## 7. Parallelization   

To enable real-time processing, thread-level parallelization was employed. The offline pipeline is parallelized over multiple nodes as well.

In the online pipeline, three classes of threads are created: one master thread, eight image acquisition threads and many *Cheetah* worker threads. The master thread creates all other threads. As soon as an image is completed by the image acquisition threads, the master thread spawns a *Cheetah* worker thread and initiates spot finding. Each of the eight image acquisition threads is responsible for reading raw images from a corresponding MPCCD module as soon as the images become available. The first image acquisition thread is also responsible for memory allocation.

To minimize the overheads of thread synchronization, an efficient algorithm based on the ‘producer–consumer’ design pattern was devised. An array with eight elements is shared among all threads. Each element keeps track of the latest tag number already acquired for the corresponding detector module. An image acquisition thread (except for the first thread) waits until the first element reaches the tag number it next reads. This ensures that memory allocation has been completed by the first thread. Then the thread reads the image into the buffer and increments its field in the array. The master thread can dispatch a spot finding thread for an image if and only if all eight fields of the array exceed its tag number. Since each field is atomically incremented by a thread and read by others, no mutex lock is necessary. In the actual implementation, complications arose because images must be skipped when the API fails or processing takes too long. To simplify memory management, a single mutex lock was introduced. Fortunately, it did not affect the performance.

For offline processing, the master node, which runs the *Cheetah Dispatcher* GUI, submits three *Cheetah* jobs for a run. Each job occupies a node (12 threads) and processes a subset of images in a run. For time-resolved experiments, two jobs are submitted: one job handles dark (non-excited) patterns and the other processes light (excited) patterns. Each node works independently. The master node monitors the progress of each job by repeatedly reading the log files. Although direct messaging through MPI (Gropp *et al.*, 1996 ▸) might be more effective, we chose file-based communication because of its simplicity and flexibility. When a worker job or even the master node crashes, the recovery is straightforward: resubmitting the affected job or re-launching the master program is sufficient. Another benefit is that we can dynamically allocate worker nodes on demand.

When reprocessing *Cheetah* outputs in batch, GNU *parallel* (Tange, 2011 ▸) was useful. GNU *parallel* reads a list of target HDF5 files and builds a command line for each target. By specifying worker nodes allocated by the queue system in the --sshloginfile option, each target can be processed on different nodes. For CPU-bound tasks such as integration, this scaled well with the number of nodes.

## 8. Parameter optimizations   

Many parameters affect the processing. We used the fixed parameters for hit finding in *Cheetah* for all data sets and did not change them. In contrast, we optimized the *CrystFEL* parameters for each dataset by a grid search.

There is a trade-off between false positives and false negatives. For hit finding in *Cheetah*, we chose permissive criteria because modest numbers of false positives are more acceptable than discarding many diffraction patterns as false negatives. After the image conversion described above, a single set of *Cheetah* parameters could be used regardless of the photon energy. Although different injection methods produced various degrees of background scattering, the same set of parameters worked well. For spot finding in *CrystFEL*, however, false positives and false negatives are both harmful for successful indexing. Thus, parameter optimization is indispensable.

In the offline pipeline, we usually run *indexamajig*, *CrystFEL*’s indexing and integration module, without providing lattice constants. This keeps users aware of unexpected changes in the crystal form. Otherwise, *CrystFEL* will reject lattices that do not match the provided cell parameters. Then, *CrystFEL* jobs are submitted again with the confirmed cell parameters (Fig. 2 ▸, manual steps).

After the completion of the *CrystFEL* jobs, users are encouraged to examine the distribution of cell parameters using *cell_explorer.* Low index rates and broad skewed distributions of cell parameters typically result from bad spot finding parameters (--min-snr, --min-gradient and --threshold) and errors in the beam centre and the camera distance. In this case, users should test *indexamajig* on a subset (several hundreds) of images with various parameters and choose the parameters that lead to the highest index rate. The beam centre can be optimized by the *detector-shift* script distributed with *CrystFEL*. Finally, the detector metrology is refined by running *geoptimiser* (Yefanov *et al.*, 2015 ▸) on all indexed images. Although these procedures are not automated, script templates are provided with the pipeline. These optimizations typically increase the indexing rate by 5–10% and improve the resolution at which CC_1/2_ falls to 0.5, by 0.1–0.2 Å. For example, a 1% error (0.5 mm) in the detector distance sometimes reduces the index rate by more than 5%. The importance of geometry optimizations is also reported by Nass *et al.* (2016 ▸). The best parameters are similar for data sets collected during the same beamtime at the same wavelength. Thus, we often optimize the parameters on a high-resolution data set with sufficient images and then apply these parameters to lower-resolution smaller data sets.

When the user is confident about the best parameters, the default parameters in the pipeline can be overridden using the --crystfel-args option of the *Cheetah Dispatcher* GUI. This feature is especially useful in time-resolved experiments, in which data sets of a protein with known cell constants are collected using a fixed geometry for a long time. Here, the best parameters are determined from a few calibration runs at the start of the beamtime and used in the offline pipeline throughout subsequent data collection. The users do not have to run *CrystFEL* again; instead, the stream files from the pipeline can be merged (Fig. 2 ▸, dotted line) and difference maps are generated every few runs. Thus one can examine how the features develop in the map and decide when to move to the next time point.

## 9. Example   

The offline and online pipelines were first introduced in November 2014 and July 2015, respectively, and have been continuously developed. Since then, about 90% of the SFX experiments at SACLA have been conducted with our pipelines. These experiments include time-resolved studies (Nango *et al.*, in preparation) and experimental phasing (Nakane *et al.*, 2015 ▸; Fukuda *et al.*, 2016 ▸). The experimental setups were also diverse; the chamber atmosphere was air or helium with or without water vapour. The injection media were liquid, grease or lipidic cubic phase. Our pipelines worked well with every combination. Here we provide an example from our beamtime in October 2015.

During a four-day beamtime, more than 5 100 000 images in 997 runs were collected at 30 Hz, for about 20 targets from several research groups. This amounted to approximately 41 TB of raw data. Real-time analysis was performed throughout the beamtime. The analysis results were displayed on the beamline computer to guide experiments, with less than a few seconds of latency. The CPU usage of the online analysis server was about 800–1000%. Since this server has 32 logical cores (two Intel Xeon E5-2667 v2 at 3.30 GHz with hyperthreading), the maximum usage corresponds to 3200%. Thus, the system can probably work at a 60 Hz repetition rate, which is expected to be available in the near future at SACLA.

The offline pipeline was executed on 16 HPC nodes (two Intel Xeon X5690 at 3.47 GHz, thus 12 threads each) in the automatic mode. Depending on the hit rate and HPC loads, the processing time from the end of a run to the completion of the pipeline varied from 3 to 20 min. Images were written to a temporary work area on a high-performance distributed filesystem, Lustre (http://lustre.org/), and subsequently moved to the long-term storage. The output of hit images from the pipeline was only 3.2 TB in total, which was less than 8% of the raw data. The size of each data set ranged from tens to hundreds of gigabytes, and was comparable to the amount collected during a typical synchrotron beamtime. Thus, the output was easily transferable by a portable disk drive or over the internet.

After the completion of the pipeline, researchers manually executed *CrystFEL* on hit images from *Cheetah* using optimized parameters. Since the output contained only hit images, the processing was efficient. In this way, most data sets were reduced to an MTZ file, and initial molecular replacement or experimental phasing solutions were obtained during the beamtime.

We note that the feedback from the pipeline allowed efficient and flexible data collection. At SACLA, we typically take turns collecting data from several targets. While a sample is being injected, the next sample is being prepared. When the hit rate of the current sample falls, the injection is stopped and the next sample is mounted on the injector. Once the number of indexed frames reaches a planned number (*e.g.* 10 000), data collection for another target is started while the data set is merged and evaluated. If the quality is unsatisfactory, that is, CC_1/2_ is bad, the difference map is noisy or phasing is unsuccessful, then more images from the first target are collected later in the beamtime. Without rapid feedback and automated image processing by the pipeline, the decision making process would be delayed and tedious.

## 10. Conclusion   

In summary, our data processing pipeline at SACLA enables real-time feedback on data collection and rapid structure solution on site. Although the user responses have been positive, there are two issues for future developments.

First, further automation is necessary, especially for parameter optimizations. Currently, at least one crystallographer who is familiar with SFX data collection and analysis must assist users during beamtime. We are planning to provide detailed tutorials and automated scripts, so that new users can quickly learn how to process their data by themselves.

Secondly, we are working to reduce the latency of analyses, for example by indexing in parallel with data collection using spot lists from the online version of *Cheetah*. We are also testing the pipeline on the Mini-K, a massively parallel system based on the SPARC architecture common to the K supercomputer (Yokokawa *et al.*, 2011 ▸).

## 11. Program availability   

Modified versions of *Cheetah* and *CrystFEL* (*hdfsee*) are available in source code on the authors’ GitHub repository (https://github.com/biochem-fan/). To interact with the SACLA DAQ system, the programs must be linked with the SACLA API, which is available on data analysis servers (online) and SACLA HPC nodes (offline). The distributed nature of the *git* version control system enables flexible development, as SACLA-specific modifications are first made on the forked version in the authors’ repository. After testing, pull requests are sent to the official repository for merging.
